# Mechanical control of molecular machines at an air–water interface: manipulation of molecular pliers, paddles

**DOI:** 10.1080/14686996.2024.2334667

**Published:** 2024-03-27

**Authors:** Taizo Mori

**Affiliations:** Institute for Chemical Research (ICR), Kyoto University, Uji, Kyoto, Japan

**Keywords:** Molecular machine, molecular conformation, air–water interface, Langmuir-Blodgett, monolayer

## Abstract

Many artificial molecular machines have been synthesized, and various functions have been expressed by changing their molecular conformations. However, their structures are still simple compared with those of biomolecular machines, and more energy is required to control them. To design artificial molecular machines with more complex structures and higher functionality, it is necessary to combine molecular machines with simple movements such as components. This means that the motion of individual molecular machines must be precisely controlled and observed in various environments. At the air – water interface, the molecular orientation and conformation can be controlled with little energy as thermal fluctuations. We designed various molecular machines and controlled them using mechanical stimuli at the air – water interface. We also controlled the transfer of forces to the molecular machines in various lipid matrices. In this review, we describe molecular pliers with amphiphilic binaphthyl, molecular paddles with binuclear platinum complexes, and molecular rotors with julolidine and BODIPY that exhibit twisted intramolecular charge transfer.

## IMPACT STATEMENT

This review discusses the dependence of the behaviour of molecular machines around their environment through the mechanically control of simple molecular machines at the air – water interface.

## GRAPHICAL ABSTRACT

**Figure:**
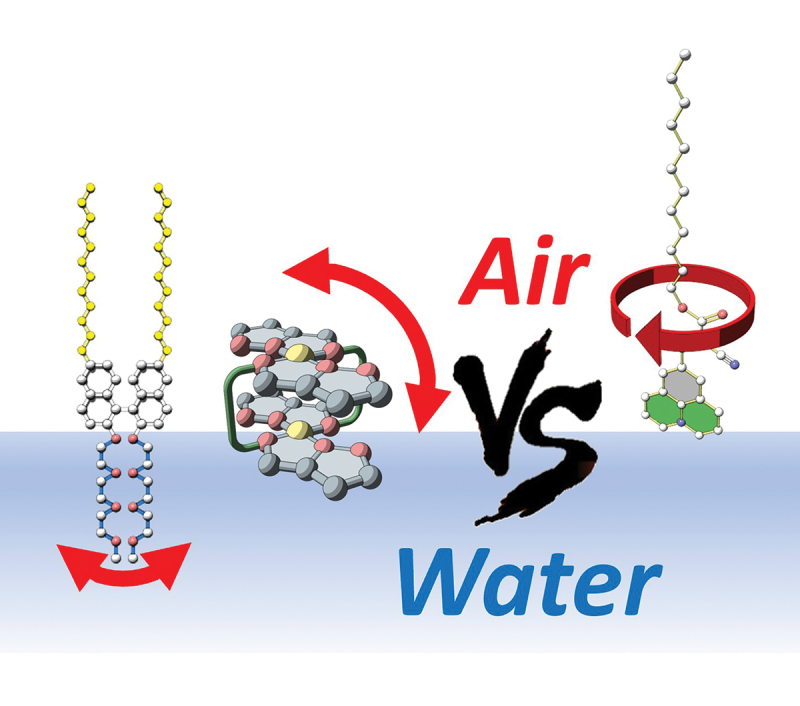

## 1. Introduction

Molecular functions are nano-level phenomena, such as molecular motions and chemical reactions, that can be used as macroscopic phenomena by hierarchically passing through multiple scales. In living organisms, various functions are expressed by the concerted interactions of various molecules, and biological activities are maintained by such interactions. It is necessary to fully understand not only individual molecules but also molecular interactions and self-assembly processes to control molecular functions.

The development of supramolecular chemistry, which evolved from host – guest molecular chemistry, has resulted in various approaches to control the molecular assembly states [1–3]. In addition, artificial molecular machines with various functions have been designed and synthesized based on the supramolecular interactions. However, the molecular structures of artificial molecular machines are not easy to synthesize because of their complexity, and the realized functions are simpler than those of biological molecular machines. Furthermore, artificial molecular machines require more power and energy to control than biological molecular machines [4–6]. Artificial molecular machines primarily use heat, light, and redox reactions as external stimuli, which correspond to a force of approximately nN per molecule. Conversely, in biological molecular machines, the force is controlled by a weak force of approximately pN by skillful use of thermal fluctuation [7,8]. Artificial molecular machines can only realize simple motions such as translation and rotation, whereas, in biomolecular machines, simple motions are coordinated among molecules to express complex functions. Artificial molecular machines can also realize advanced functions, such as biological molecular machines, by combining several molecules as parts and making them work in concert. Therefore, it is necessary to investigate in detail the operating principles of artificial molecular machines and the molecular conformational changes that form the basis of their motions. Mechanical stimuli are common external stimuli; however, their response to molecules is largely unknown. Many molecular machines output their work in terms of mechanical energy. In living organisms, the mechanical energy generated by molecular motors is used to transport molecules and change their molecular conformations to perform their functions. Although it is important to understand the mechanical response of both artificial and biological molecular machines, it is still unclear how much force and in which direction the conformational change occurs when force is applied.

Molecular machines have been investigated mainly because of their motion in solution or in bulk. A two-dimensional interface with fewer degrees of freedom is preferable to a three-dimensional space for observing the motion of molecular machines. At solid interfaces, molecular machines were observed and their motions controlled by scanning electron microscopy (SEM) and atomic force microscopy (AFM) [9,10]. Molecular machines vapor-deposited on metal surfaces have been observed by SEM, and their molecular conformational changes and motions have been controlled by the mechanical actions of the probe and tunneling currents. More recently, using molecules deposited on carbon nanohorns or encapsulated in nanotubes, three-dimensional molecular motion and conformational changes can be observed in real time using transmission electron microscopy. Although these techniques allow direct observation of molecules, they are performed in vacuum and at the solid interface, which is very different from the environment in which molecules are normally used, such as in biological organisms. As examples of the application of molecular machines at interfaces, researchers have reported the use of cantilevers in atomic force microscopes to control the motion of molecular machines [11] and the use of molecular rotors to pierce lipid bilayers [12].

The air – water interface is a suitable place to investigate the principle of operation of molecular machines because their conformation and assembly can be controlled by macroscopic motions [13,14]. The application of the air – water interface enables not only typical amphiphilic film formation and molecular machine control, but also metal organic frameworks [15] and nanoparticles [16] film formation as well as precise doping control of organic semiconductors [17]. The Langmuir – Blodgett (LB) system can continuously control molecular assembly and conformation at the air – water interface by compressing and expanding the macroscopic barrier [18]. Furthermore, the orientation of the molecules at the air – water interface is controlled, and the forces on the molecules are limited to a direction parallel to the air – water interface. K. Ariga et al. used the LB system to mechanically control various molecular machines at the air – water interface [19–30]. Molecular machines comprising cyclic cyclophanes modified with steroids as side chains exhibit fluorescence at the air – water interface, where the steroid moiety captures the fluorophore in the aqueous phase during compression and releases it during expansion to quench fluorescence [19]. In addition, T. Michinobu and T. Mori et al. found that the binding constants of cyclic amines with cholesterol side chains to amino acids and nucleic acids in the aqueous phase change depending on the degree of compression of the molecular machine [20,21]. Thus, at the air – water interface, it is possible to regulate the molecular conformation that exhibits optimal molecular functions using mechanical stimuli such as compression and expansion.

At the air – water interface, the motion of molecular machines can be continuously changed using mechanical stimulation. However, understanding the operation of the aforementioned molecular machines is difficult because of their complex structures. To combine molecular machines to perform more complex functions, it is necessary to design molecular machines with simple structures, observe their movements under various stimuli and environments, including the air – water interface, and accumulate knowledge of them. Conversely, there are not many methods for observing the motion of molecular machines at the air – water interface. In particular, in many cases, sufficient signal intensity for detection cannot be obtained from the monolayer for in situ observation. Molecular emission, such as fluorescence, can provide sufficient intensity for detection even in a monolayer. However, molecular machines do not always show fluoresce, and their movement is not always accompanied by a change in fluorescence. Fluorescent molecules can be modified as probes; however, the possibility of interfering with the original molecular motion must be considered. Other methods include ellipsometry and nonlinear optics, such as second harmonic and sum frequency generation. However, ellipsometry is a method for molecular assembly and not for tracking molecular motion, and nonlinear optics is not an easy measurement system to constructed.

With a transfer film, it is possible to obtain sufficient signal intensity for measurements by stacking the layers, which allows not only optical spectral measurements but also AFM and electron microscopy observations. However, the possibility that molecular orientation or conformational changes may have occurred during transfer rather than at the air – water interface should be considered.

**Figure 1.**
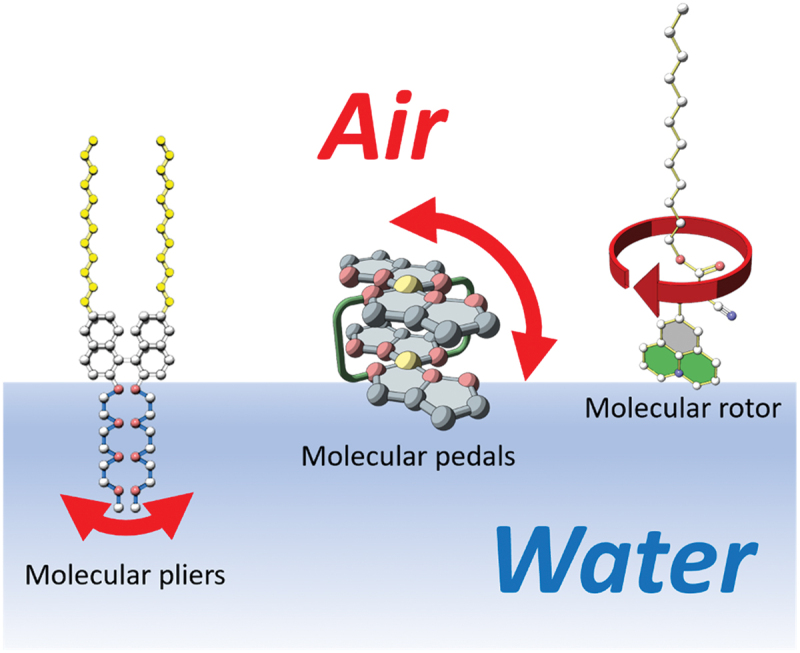
Schematic illustration of operation of molecular machines: molecular pliers (bending), molecular paddle (flip-flop), and molecular rotor (rotation) at the air−water interface.

In this review, we focus on molecular machines with simple structures and describe examples of their mechanical control at the air – water interface [22–31]. As simple molecular machines, we introduce molecular pliers that control opening and closing [24], molecular paddles that change orientations [26], and molecular rotors [27,28] that rotate intramolecularly (Figure 1).

## 2. Molecular machine at air – interface

### 2.1. Molecular pliers of axially chiral binaphthyl molecule

**Figure 2.**
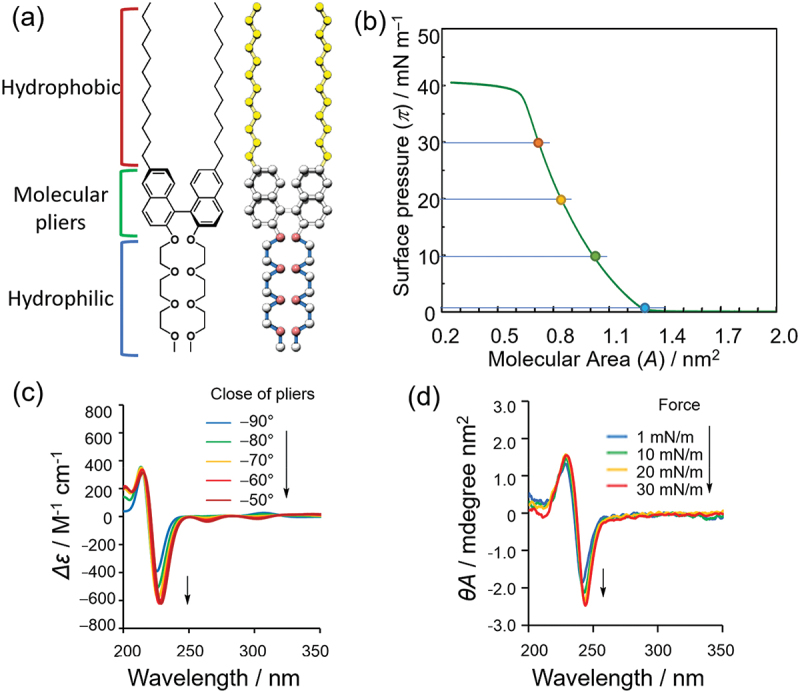
(a) Structure and schematic illustration of amphiphilic binaphthyl as molecular pliers. (b) *π*–*A* isomer of molecular pliers. (c) Time-domain DFT-based CD spectral simulations of model binaphthyl structure and (d) CD spectra of molecular pliers transferred at different surface pressures multiplied by molecular area.

It is desirable to be able to measure the process of continuous conformational change to understand the mechanical behavior of molecular machines at the air – water interface. An axially chiral binaphthyl molecule exhibits plier-like continuous changes in the dihedral angle (*ϕ*) between two naphthalene rings [32]. Because of the ease of chemical modification, binaphthyl molecules have been designed as various molecular machines, and the dihedral angles have been controlled using various stimuli. The circular dichroism (CD) of the binaphthyl molecule changes with the dihedral angle, and the signal is sufficiently strong to be detected, even in monolayers. D. Ishikawa et al. synthesized amphiphilic binaphthyl with dodecyl side chains as the hydrophobic moiety and triethylene glycol as the hydrophilic moiety as molecular pliers (Figure 2(a)) [24].

The amphiphilic binaphthyl was spread at the air – water interface to measure the surface pressure – molecular area (*π*–*A*) isotherm, which exhibited a well-defined monolayer that collapsed at approximately 40 mN m^−1^ (Figure 2(b)). The monolayers were then transferred to quartz substrates using the LB method at different surface pressures, and the CD spectrum of the transferred film was measured. Consequently, the peak intensity at approximately 240 nm originating from the dihedral angle increased with transfer pressure (Figure 2(c)). It was also observed that the peak intensity increased or decreased with repeated compression and expansion of the barrier. Density functional theory (DFT) calculations for the binaphthyl molecule also confirmed that the intensity of the CD peak increased as the dihedral angle decreased (Figure 2(d)). In other words, the binaphthyl molecule, which is a molecular plier, is closed and opened by compression and expansion of the monolayer, respectively.

The correlation between the energy applied to the monolayer and the degree of opening/closing was estimated using the *π–A* isotherm and DFT and molecular dynamics (MD) calculations. As an applied total energy of a monolayer compression can be calculated from the integration of the *π*–*A* isotherm, an energy equivalent to 1 kcal mol^−1^ was applied to the monolayer of binaphthyl molecules at a surface pressure of 30 mN m^−1^. DFT calculations were performed on a model molecule consisting only of binaphthyl molecules to estimate the relationship between the intensity of the CD spectrum and the dihedral angle and the energy required to change the dihedral angle. The results show that the dihedral angle of the binaphthyl molecule closes from 90° to 80° by compression and that the energy required is approximately 1 kcal mol^−1^, which is in agreement with the experimental results. Furthermore, MD calculations of the relationship between the molecular area and dihedral angle show that the dihedral angle opens to approximately 90° before the surface pressure increases in the *π*–*A* isotherms and closes to 80° at 0.6 nm^2^ when the surface pressure reaches 30 mN m^−1^. This result is also in agreement with the experimental and DFT calculations.

In conclusion, the opening and closing of the amphiphilic binaphthyl molecular pliers can be controlled by compressing and expanding the monolayer at the air – water interface, respectively. The energy required to open and close the monolayer is as low as 1 kcal mol^−1^, which is equivalent to thermal fluctuation, and the dihedral angle changes continuously from 90° to 80° by compression and expansion. Although there are few reported cases in which the conformation of binaphthyl molecules is controlled at the air – water interface, a change in the molecular arrangement has been observed for binaphthyl molecules with carboxylic acids as hydrophilic moieties upon compression [33]. In addition, S. Akine et al. reported an attempt to control the molecular conformation of salen complexes with axial chirality at the air – water interface [31].

### 2.2. Molecular paddles of H-type binuclear Pt(II) complexes

**Figure 3.**
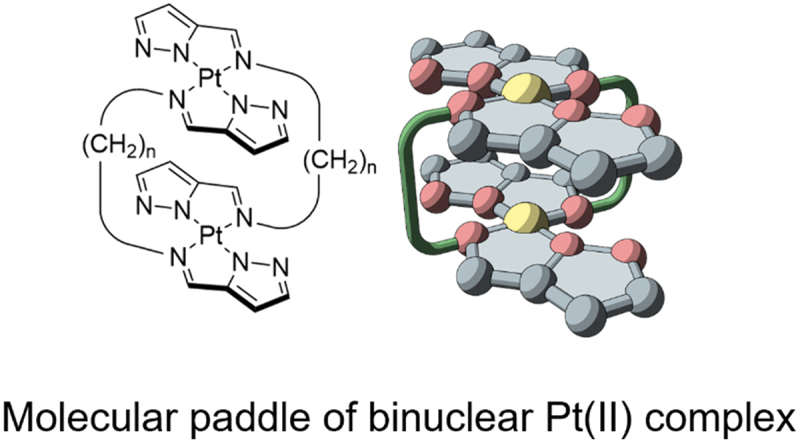
Chemical structure and schematic illustration of double-paddled binuclear Pt(II) complexes.

Amphiphilic molecules are oriented at the air – water interface such that the hydrophobic part faces the gas phase and the hydrophilic part faces the aqueous phase. J. Adachi et al. designed molecular paddles and synthesized H-type binuclear complexes with two complex planes connected by a linker to mechanically control the molecular orientation at the air – water interface (Figure 3) [26].

The H-type binuclear complexes exhibit flapping and rotational motions around the linker connecting the two complex planes [34–36]. In isotropic solvents, two complexes with long linkers can move freely, whereas those with shorter linkers have restricted motion of the complex planes and slower isomerization rates, allowing the separation of planar chirality [37,38]. At the air – water interface in an anisotropic field, even a long linker is expected to behave differently than in an isotropic solvent because of the restricted motion of the complex planes. A binuclear Pt(II) complex consisting of a pyrazole ring and forming planar tetracoordinated structures was synthesized as a molecular paddle. Pt(II) complexes with pyrazole rings as ligands exhibit phosphorescence and quenching with the formation of hydrogen bonds between the nitrogen atoms of the pyrazole ring [39]. In addition, mononuclear Pt(II) with a pyrazole ring crossed by an alkyl linker forms a monolayer at the air – water interface, but not with an imidazole system in which the nitrogen position differs [40]. In other words, H-type binuclear Pt(II) complexes consisting of pyrazole rings are suitable molecules for estimating the orientation direction of the complex plane by phosphorescence at the air – water interface.

H-type binuclear platinum complexes with alkyl chains of various lengths of *n* = 7–12 carbons as linkers and pyrazole rings as complex planes have been synthesized as molecular paddles. The nuclear magnetic resonance (NMR) results show that at *n* = 7, the isomerization progress is so slow that the *syn* and *anti* diastereomers can be identified individually, whereas, at *n* = 8 or higher, the isomerization progresses are so fast that the complex as a paddle is free to rotate. H-type binuclear platinum complexes exhibit strong phosphorescence at approximately 530 and 570 nm in nonprotic solvents, whereas the intensity decreases in ethanol, a protic solvent. DFT calculations also confirm that quenching occurs when the nitrogen atom of the pyrazole ring forms a hydrogen bond. Incidentally, the solubility of a carbon number of *n* = 9 was abnormally low, and its quantum yield was less than half that of the others. Because the synthesis yield of *n* = 9 is also low, it is thought to be prone to aggregation even though the detailed mechanism is unknown.

The H-type binuclear Pt(II) complexes were then spread at the air – water interface, and the phosphorescence spectra were simultaneously measured during compression. An optical fiber connected to an excitation light source and a detector was brought close to the air – water interface, and the emission spectrum was observed in situ. The phosphorescence intensities at 530 and 575 nm, normalized to the molecular area, are plotted on the *π–A* isotherms. The *π–A* isotherms and phosphorescence spectra exhibit different behaviors depending on the linker length.

**Figure 4.**
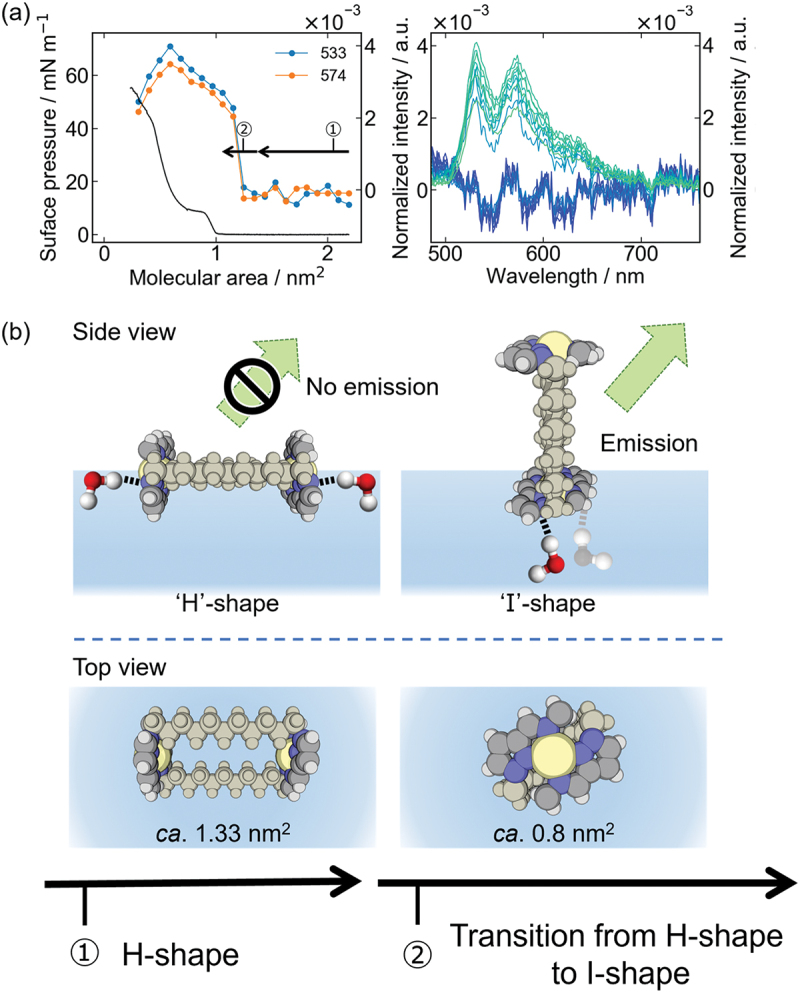
(a) Emission intensity with area per molecule (circles/lines; blue: 533 nm; orange: 574 nm) observed from monolayers of binuclear Pt(II) complex (*n* = 11). *π*–*A* isotherm is also shown as black lines. Emission spectra observed from the monolayers of the complex at different molecular areas excited at 400 nm. Molecular area reduces as it passes from the blue line to the green line (2.2 to 0.3 nm^2^ by 0.2 nm^2^). (b) Schematic illustration of changes in conformation and emission intensity of the complex at the air – water interface during mechanical compression.

At *n* = 7, no phosphorescence was observed regardless of the surface pressure, whereas, at *n* = 10, phosphorescence was always observed. Even at *n* = 9, where solubility is low, phosphorescence was observed regardless of compression. As the surface pressure increased at 0.4 nm^2^, which was smaller than calculated molecular of 0.8 nm^2^, it is assumed that the molecules aggregate three-dimensionally at the air – water interface and do not form a two-dimensional monolayer. Linker lengths *n* = 8, 11, and 12 yielded characteristic step-like *π–A* isotherms, and the phosphorescence intensity increased with compression before the surface pressure increased. The molecular area on which the surface pressure starts increased to 1.24, 1.33, and 1.36 nm^2^ for *n* = 8, 11, and 12, respectively (Figure 4(a)). Compared with the cross-sectional area of the H-shaped molecule area calculated from the X-ray diffraction (XRD) analysis results, the area increases with the length of the alkyl chain. An area of approximately 1.3 nm^2^, where the phosphorescence starts to increase, corresponds to an H-shaped area where the two complex planes are perpendicular to the air – water interface (Figure 4(b)). Conversely, 0.8 nm^2^, where the surface pressure plateaued, corresponds to the area of the complex plane, suggesting that the complex plane is reoriented parallel to the air – water interface. The orientation of the H-type binuclear Pt(II) complexes at the air – water interface was evaluated from the IR reflection absorption spectroscopy (RAS) of the LB transferred films. Linker lengths *n* = 8 and 11 showed a regular alignment of the complexes with increasing surface pressure. On the other hand, at *n* = 7 and 10, no significant difference was observed between the LB and cast films, suggesting that the complexes were arranged in a disordered manner. Combined with the previous result of quenching due to hydrogen bond formation, the following mechanism can be considered.

Immediately after spreading at the air – water interface, the two complex planes become perpendicular to the air – water interface and form an H-shape in contact with water. As the molecule is compressed, one of the complex planes touches the water surface and the other rises to the gas phase to form an I-shape at the air – water interface. In the H-shape, both complex planes are in the water phase and form hydrogen bonds with water molecules, quenches light. Conversely, in the I-shape, one of the complex planes is in contact with water and is quenched, whereas the other is in the gas phase and exhibits phosphorescence. In other words, the H-type binuclear complexes at the air – water interface exhibit phosphorescence because the orientation of the complex plane changes from perpendicular to the air – water interface to horizontal during compression.

At *n* = 7, the linker is short and the rotation of the complex plane is inhibited; therefore, the H-shape is maintained during compression, and no phosphorescence is observed. Conversely, at *n* = 10, the two complex planes are not aligned in the torsional direction and cannot form hydrogen bonds with water molecules because of steric repulsion; therefore, the H-shaped complex remains suspended in the gas phase and exhibits phosphorescence. The phosphorescence intensity decreased as the complex planes aligned and formed hydrogen bonds with water molecules because of quenching by compression.

The H-type binuclear Pt(II) complexes, which are molecular paddles, exhibit different rotational behavior between the two complex planes and the direction in which the complex planes face each other at the air – water interface, depending on the linker length. In other words, a slight structural difference in the driving site, which is the pivot point of molecular conformation and orientation change in a molecular machine, affects the molecular orientation behavior at the air – water interface.

### 2.3. Molecular rotors of julolidine and its BODIPY derivatives

In molecular pliers and paddles, the driving sites are located in both the gas and aqueous phases. T. Mori et al. demonstrated the dynamic control of the intramolecular rotation of julolidine [41–43] and BODIPY [44,45] at the air – water interface to observe the behavior of molecular machines in the gas phase. The molecular rotor is quenched by twisted intramolecular charge transfer (TICT) during intramolecular rotation and fluoresces by rotation inhibition. Therefore, the higher the viscosity of the solution, the higher the fluorescence intensity. Because of their properties, they can also be used as fluorescent probes for enzyme reactions and other applications.

**Figure 5.**
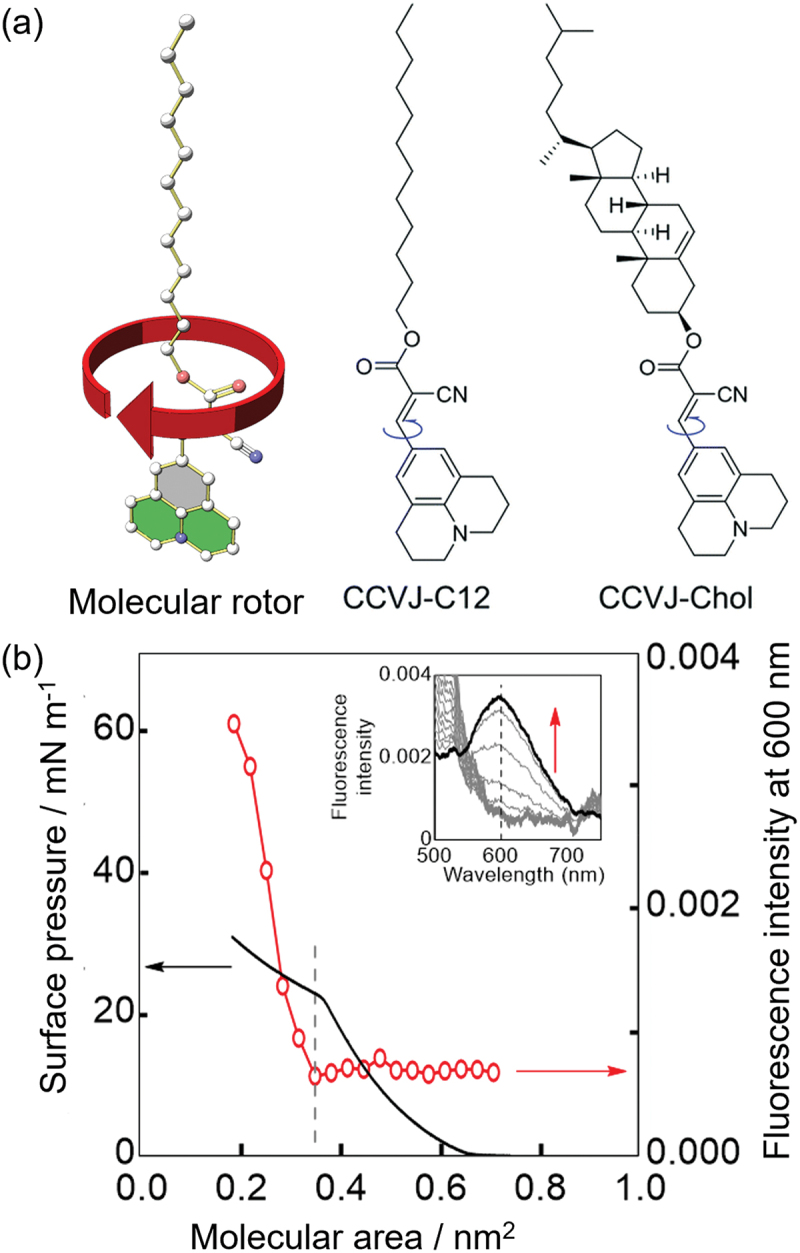
(a) Schematic illustration and chemical structures of CCVJ-C12 and CCVJ-Chol. (b) Emission intensity with area per molecule (circles/lines; 600 nm) observed from monolayers of CCVJ-C12. *π*–*A* isotherm is also shown as black lines. Emission spectra observed from the monolayers of the complex at different molecular areas excited at 450 nm. Molecular area reduces as it passes along the red arrow from 0.7 to 0.2 nm^2^ by 0.012 nm^2^.

CCVJ-C12 and CCVJ-Chol were synthesized by introducing dodecyl and cholesterol groups, which are alkyl chains, into julolidine as molecular rotors (Figure 5(a)) [27]. Both molecules exhibited fluorescence at approximately 500 nm, and the intensity increased linearly with the viscosity of the solvent. In the solid power or in condensed solution, both of CCVJ-C12 and CCVJ-Chol showed the excimer emission at 600 nm. The two molecular rotors were then spread at the air – water interface, and fluorescence was observed during compression. Although each rotor formed a monolayer, no fluorescence was observed below the collapse pressure, and fluorescence at 600 nm derived from the excimer was observed after the collapse. The collapsed films are thought to form an aggregate state similar to that of the bulk solid.

CCVJ-C12 and CCVJ-Chol exhibited no fluorescence below the collapse pressure, suggesting that intramolecular rotation in the monolayer was not inhibited. However, the fluorescence intensity may have been too weak to detect. Therefore, using the same technique as at the air – water interface, fluorescence was measured in a system in which both molecular rotors were spread on ethylene glycol, which is more viscous than water, and in cast and LB films formed on a glass substrate. Under each condition, the molecular area was adjusted to 0.6 nm^2^, which is less than the collapse pressure. Fluorescence was observed at approximately 500 nm on ethylene glycol, confirming the inhibition of intramolecular rotation. In addition, the cast and LB films exhibited fluorescence at 600 nm, originating from the excimer, indicating that the excimer was formed in the solid state regardless of the molecular area. These results indicate that the intramolecular rotation of julolidine is not inhibited by compression at the air – water interface.

Because the molecular area of the julolidine moiety is larger than that of the side chains (alkyl chains and cholesterol groups), the side chains cannot interact with each other, even as the rotor molecules approach each other by compression, and intramolecular rotation is not inhibited. Thus, intramolecular rotation could be inhibited if mechanical stimuli were transmitted to the side chains of the rotor molecules. Therefore, inhibition of the intramolecular rotation of molecular rotors has been demonstrated in lipid matrices.

## 3. Molecular machine in lipid matrix

### 3.1. Molecular rotors of BODIPY in lipid matrix

**Figure 6.**
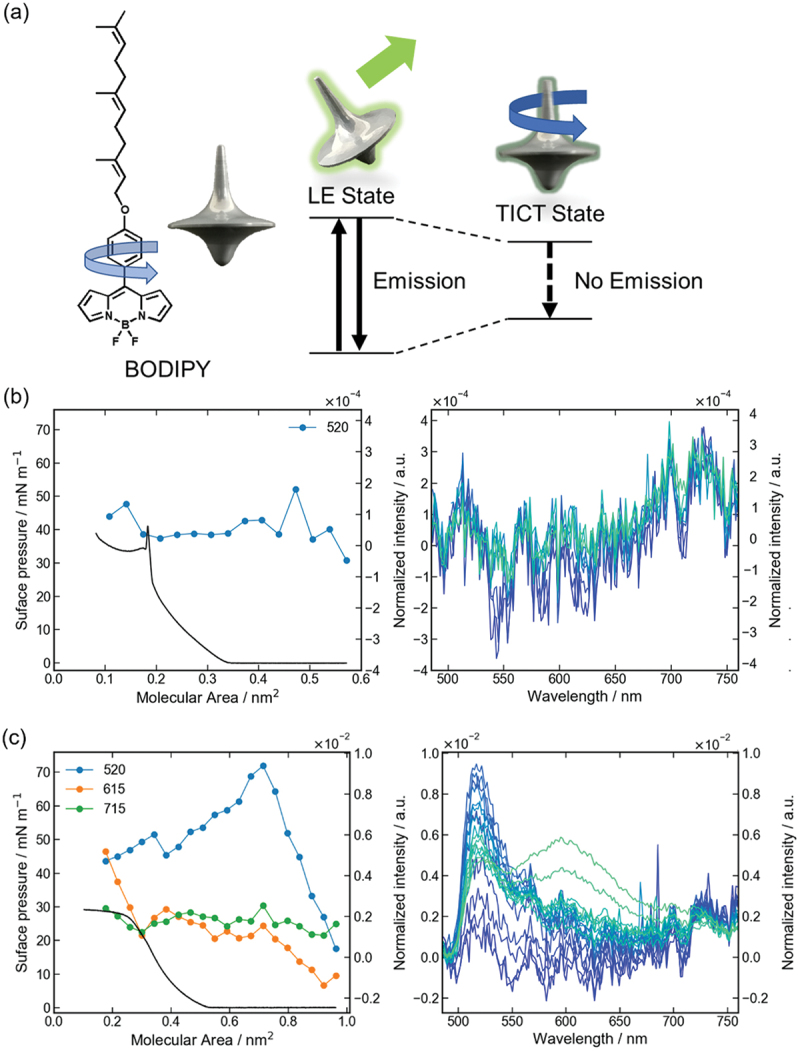
(a) Chemical structure and energy diagram of the molecular rotor, BODIPY. In its locally excited (LE) state, BODIPY does not rotate and exhibits fluorescence emission. In its TICT state, BODIPY undergoes intramolecular rotation, and no fluorescence emission occurs. Variation in fluorescence intensity with area per molecule (circles connected lines) observed from mixed monolayers of BODIPY and lipids: (b) stearic acid and (c) oleic acid. *π*–*A* isotherms are also shown as black lines. BODIPY/each matrix at molar ratios of 1/10. Fluorescence spectra observed from the monolayer of BODIPY at different molecular areas excited at 400 nm. Molecular area reduces as it passes from the blue line to the green line.Spectra observed from the monolayer of BODIPY at different molecular areas excited at 400 nm. Molecular area becomes smaller passing from the blue line to the green line from 0.6 to 0.1 nm^2^ by 0.05 nm^2^ and (c) from 1.0 to 0.2 nm^2^ by 0.05 nm^2^ (b).

Because the julolidine molecule has a weak fluorescence intensity, it has been used as a molecular rotor with a masoner side chain introduced into BODIPY, which has a stronger fluorescence (Figure 6(a)) [28]. BODIPY exhibited fluorescence at approximately 520 nm, and as with other molecular rotors, the intensity increased with solvent viscosity.

BODIPY was spread on the air – water interface, and its fluorescence spectrum was measured during compression. In the *π*–*A* isotherms, the surface pressure started to increase from a molecular area smaller than 0.55 nm^2^, which was the molecular area for edge-on orientation of the long axis of the BODIPY moiety at the air – water interface, suggesting the formation of three-dimensional aggregates rather than monolayers. Correspondingly, in the fluorescence spectrum, the fluorescence around 520 nm decreased with compression, and the fluorescence intensity at 615 nm, derived from the excimer, and at 720 nm, derived from aggregates of two or more molecules, increased. Although BODIPY also fluoresces at approximately 520 nm at the air – water interface, the *π*–*A* isotherms and fluorescence measurements suggest that the intramolecular rotation of BODIPY is inhibited by the aggregation of BODIPY molecules and not by the propagation of mechanical stimuli to BODIPY.

The inhibition of intramolecular rotation of BODIPY was then confirmed in various lipid matrices. Cholesteryl acetate, linear alkyl stearate, and unsaturated oleic acid were used as lipids, and DPPC (dipalmitoylphosphatidylcholine) and DOPC (dioleoylphosphatidylcholine) were used as phospholipids [28]. This review will briefly focus on a mixed monolayer containing rigid stearic acid and soft oleic acid.

The mechanical properties of the membrane mixed with BODIPY and lipids were evaluated using *π–A* isotherms. The limiting molecular area of BODIPY in lipids was compared with the molecular area calculated from *π*–*A* isotherms with pure BODIPY. In stearic acid, the limiting molecular area of BODIPY was smaller than the molecular area of pure BODIPY, whereas, in oleic acid, it increased significantly. The compression modulus, which corresponds to the rigidity of the monolayer, is defined as -*A*·*dπ*/*dA* from *π–A* isotherms. The compression moduli of lipids and mixed monolayers were compared, and the results showed that the compression modulus of stearic acid decreased with the addition of BODIPY, whereas it changed less with oleic acid. Rigid stearic acid, which showed a decrease in the limiting molecular cross-section and compression modulus, is assumed to have good miscibility with BODIY and poor miscibility with softer oleic acid.

The fluorescence behavior of BODIPY at the air – water interface depends on its miscibility with the lipid matrices. In rigid stearic acid with better miscibility, the intramolecular rotation of BODIPY was not inhibited and no fluorescence was observed (Figure 6(b)). In soft oleic acid, the fluorescence intensity at approximately 520 nm increased from the molecular area before the surface pressure increased (Figure 6(c)). The fluorescence intensity at 520 nm decreased from approximately 0.7 nm^2^ and was replaced by an increase in fluorescence at 615 nm, derived from the excimer. This suggests that the intramolecular rotation of BODIPY in the softer oleic acid is inhibited, even before the monolayer is formed, and that the molecules begin to aggregate and form an excimer because of poor miscibility upon further compression. In cholesteryl acetate, DPPC, and DOPC, the intramolecular rotation of BODIPY also depended on their mechanical properties and miscibility with BODIPY [28].

### 3.2. Molecular machine of binaphthyl derivatives in lipid matrix

**Figure 7.**
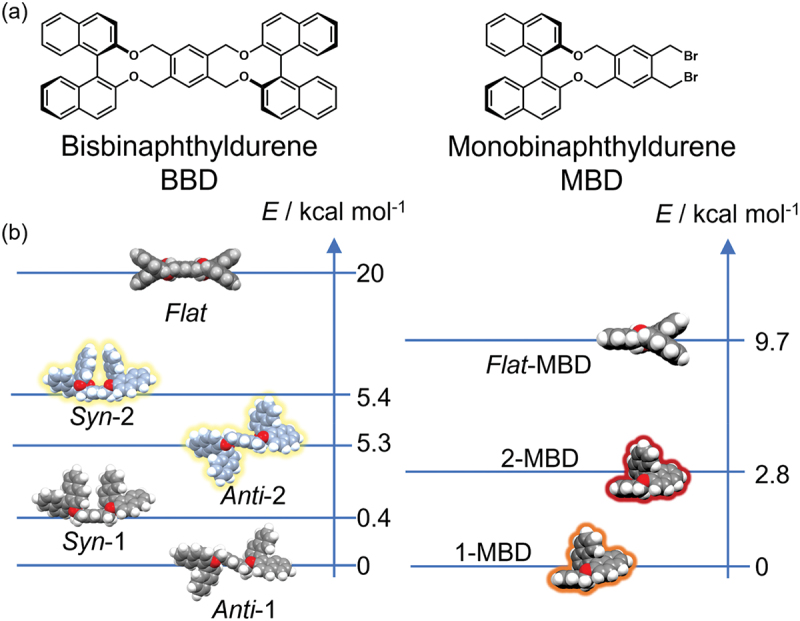
(a) Chemical structures of BBD and MBD. (b) Relative energy of BBD and MBD conformers.

The molecular conformations of binaphthyl molecules were also controlled in lipid matrices. M. Ishii et al. formed monolayers of MBD [30], a binaphthyl molecule cross-linked with durene, and its dimer, BBD [29], mixed with various lipid matrices at the air – water interface and mechanically controlled their molecular conformations (Figure 7). DFT calculations revealed that MBD and BBD have three main conformers: the most stable 1-conformers, 2-conformers with energies of a small kcal mol^−1^, and unstable *flat* conformers. The dihedral angle of 1-conformers (|*ϕ*| = 64º) is smaller than that of 2-conformers (|*ϕ*| = 89º). The *flat* conformer is stable on metal surfaces, whereas, in solution, 1- and 2-conformers have been identified by NMR. Pure MBD and BBD do not form monolayers at the air – water interface, and their molecular conformation is unchanged from that of 1-conformer by surface pressure.

**Figure 8.**
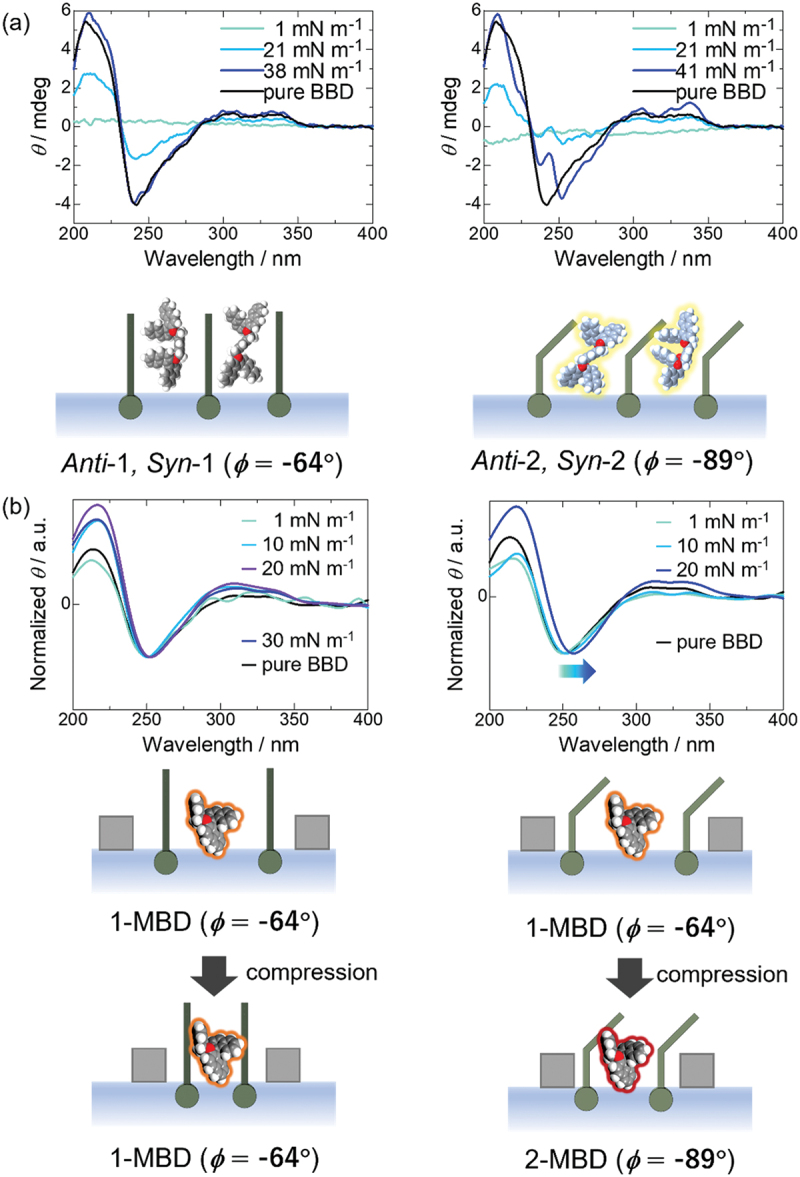
*π*–*A* isotherms of lipids (stearic acid, oleic acid) and mixtures with BBD (a) and MBD, (b) and CD spectra of mixed monolayers transferred at several different surface pressures. CD spectra of BBD and MBD films transferred at 40 and 41 mN m-1, respectively. BBD/matrix molar ratios are 1/2, MBD/matrix molar ratios are 1/1. CD intensities are normalized to their minima to clarify the shift of the peaks (b).

As lipid matrices, rigid stearic acid was used as a saturated linear alkyl, and both soft *trans* elaidic acid and *cis* oleic acid were used as unsaturated. The compression moduli of the lipid matrices were compared with those of the mixed mixture with MBD and BB. The compression modulus of the rigid stearic acid mixture increased, whereas that of the softer elaidic acid and oleic acid decreased slightly. This suggested that the miscibility of MBD and BBD with lipids was better with unsaturated elaidic acid and oleic acid than with stearic acid. The molecular conformation of the binaphthyl molecule was then examined using CD spectroscopy of LB films transferred from the mixed monolayers at specific surface pressures. The results showed that the molecular conformation of BBD remained unchanged from the 1-conformer in rigid stearic acid, whereas it changed to the 2-conformer in soft elaidic acid and oleic acid (Figure 8(a)). However, the conformation of BBD remained unchanged under surface pressure in any lipid. Similar to BBD, the molecular conformation of MBD remained unchanged from that of 1-MBD in rigid stearic acid. In contrast, in softer elaidic acid and oleic acid, the conformation of MBD changed from 1-MBD to 2-MBD with increasing surface pressure (Figure 8(b)). The difference in the mechanical response between MBD and BBD may be due to the hydrophilic properties of the C – Br bonds of durene in MBD and their different miscibility with lipids.

The force transfer of the xylose-derived molecular machine, where pyrene was introduced as a fluorescent moiety, was also dependent on its miscibility with lipids [20]. The conformation of the xylose-derived molecular machine was mechanically controlled by its miscibility with rigid lipids.

In another example, the CD spectra of the 2,2′-methyl-substituted binaphthyl molecule and DPPC and other lipid mixtures were found to invert from negative to positive at approximately 230 nm upon compression [25]. This result suggests that the dihedral angle of the naphthalene rings of the binaphthyl molecule changes from the cirsoid conformation, where the angle is less than 90°, to the transit conformation, where the angle is greater than 90°. This conformational change was observed even after repeated compression and expansion, regardless of the lipid variety. Optical microscopy, AFM, and XRD analysis confirmed the formation of binaphthyl molecule microcrystals in the lipid matrix upon compression at the air – water interface. In general, diastereomeric binaphthyl molecules have a cisoid conformation with a dihedral angle less than 90° in solution and a transoid conformation with an angle greater than 90° in the crystals. Because these microcrystals disappear with the expansion of the mixed membrane, the lipid matrices function as a solvent for the binaphthyl molecule at the air – water interface. These results confirm that the crystallization and dissolution of binaphthyl molecules in lipid matrices are controlled by compression and expansion, respectively, resulting in a conformational change from cisoid to transoid.

## 4. Summary

In summary, we will discuss the behavior of molecular machines at the air – water interface, including examples of molecular machines that could not be presented in detail in this review (Tables 1 and 2).

**Table 1.** Motion and controllability of molecular machines at the air – water interface.

Molecular machine	Motion	Driven parts	Controllability
Steroid armed cyclophane [19]	Catch and release	Contact with water	Continuous and cyclic
Cholesterol armed cyclic amine [20,21]	Molecular recognition	Contact with water	Continuous
Molecular pliers [24]	Open-closed	Contact with water	Continuous and cyclic
Molecular paddles [26]	Submarine emission	Contact with water	Orientation change
Molecular rotor [27,28]	Intramolecular rotation	At gas phase	No control

**Table 2.** Motion of molecular machines in lipid matrices at the air – water interface.

Molecular machine	Motion	Controllable lipid
Molecular rotor [28]	Intramolecular rotation	Rigid and poor solubility
Pyrene armed xylose [23]	Excimer formation	Rigid and good solubility
Bisbinaphthyl-durene [29,30]	Open-closed	Soft and good solubility

Amphiphilic binaphthyl as molecular pliers can be controlled to open and close by compression and expansion, respectively, because the driving site, a rigid naphthalene ring, is close to the aqueous phase (Table 1) [24]. In the cyclophane molecular machine, the driving site is also a rigid steroid molecule located near the aqueous phase, which facilitates the transfer of force to the molecule and allows repeated opening and closing of the steroid moiety by compression and expansion, respectively (Table 1) [19]. In the molecular recognition of cyclic amine derivatives, the cyclic amine driving site is also close to the aqueous phase and the side chain is rigid cholesterol, which facilitates force transfer (Table 1) [20,21]. Consequently, it is expected that force transfer is easier for a molecular machine with a rigid driving site located near the aqueous phase. The energy required to control the molecular machine consisting of cyclophane and cyclic amine is estimated to be a few kcal mol^−1^, which is the same as that of binaphthyl molecules.

Molecular paddles with platinum binuclear complexes have rigid pyrazole rings, which are the driving sites, located at the air – water interface (Table 1) [26]. Although the behavior of the molecular paddle at the air – water interface varies depending on the alkyl chain length of the linker, it exhibits the following submarine emission: At the air – water interface, the two pyrazole rings of the molecular paddle are quenched because they both contact the aqueous phase and form hydrogen bonds. After applying a lateral force to the molecular paddle, the orientation changes, and one of the pyrazole rings contacts the aqueous phase, whereas the other floats to the gas phase, indicating phosphorescence.

In molecular rotors, the side chains, which are the driving moieties, are in the gas phase with a high degree of freedom, and the molecular area of the side chains is small compared with that of the rotor moiety. Therefore, the side chains cannot interact with each other, and intramolecular rotation is not inhibited, even by compression (Table 1) [27,28]. In lipids, intramolecular rotation depends on the mechanical properties and miscibility of the lipids. In lipids with rigid saturated alkyl side chains miscible with the molecular rotor, intramolecular rotation is not inhibited, as in the case of pure molecular rotors (Table 2) [28]. Conversely, in the case of lipids with soft unsaturated alkyl side chains with poor miscibility, intramolecular rotation is inhibited; however, aggregates, such as an excimer, are formed by compression.

The xylose molecular machine with pyrene is in contact with the air – water interface; however, because of the softness of xylose, the force is not transferred, and the conformation does not change during compression. In the lipid, which is rigid and miscible with xylose, force is transferred to the xylose, its conformation is changed by compression, and pyrene forms an excimer (Table 2) [23].

The conformation of BBD and MBD, where a binaphthyl molecule is cross-linked with durene, can be controlled using mechanical stimuli in soft and well-miscible lipids but not in poorly miscible and hard lipids (Table 2) [29,30]. Because MBD and BBD do not form two-dimensional monolayers but three-dimensional aggregates by themselves, it is assumed that their molecular conformations are moderately dispersed and modified in lipids with better miscibility.

As described above, the motion of molecular machines depends on the interactions between molecules and their environment. It will be necessary to clarify the behavior of different molecular machines in different environments, such as in lipid bilayers consisting of vesicles and cell membranes, to realize this in artificial molecular machines. It is then expected that it will be possible to combine different artificial molecular machines to design molecular recognition and catalytic functions for specific purposes.
